# Impact of the Use of a Rapid Diagnostic Test for Visceral Leishmaniasis on Clinical Practice in Ethiopia: A Retrospective Study

**DOI:** 10.1371/journal.pntd.0003738

**Published:** 2015-05-12

**Authors:** Ermias Diro, Lutgarde Lynen, Mahlet Assefa, Yegnasew Takele, Bewketu Mengesha, Emebet Adem, Rezika Mohammed, Robert Kimutai, Asrat Hailu, Marleen Boelaert, Johan van Griensven

**Affiliations:** 1 University of Gondar, Gondar, Ethiopia; 2 Institute of Tropical Medicine, Antwerp, Belgium; 3 Yale University, Hartford, Connecticut, United States of America; 4 Drugs for Neglected Diseases initiative (DNDi) Africa, Nairobi, Kenya; 5 Addis Ababa University, Addis Ababa, Ethiopia; Royal Tropical Institute, NETHERLANDS

## Abstract

**Background:**

Diagnostic guidelines for Visceral Leishmaniasis (VL) in the East African region are complex. Patients meeting the VL clinical case definition should be tested by rK39 rapid diagnostic test (RDT) followed by the Direct Agglutination Test (DAT) or tissue aspiration if RDT-negative. Otherwise, RDT-positive patients should be started on VL treatment. We evaluated how this guideline is adhered to by assessing the routine clinical practice in a university hospital in North-West Ethiopia.

**Methods:**

Retrospective record analysis was done for all patients who had an rK39-RDT done at University of Gondar (UoG) Hospital between June 2012 and June 2013. We described the diagnostic work-up performed and the proportion initiated on VL treatment by test result.

**Results/Findings:**

From a total of 928 patients tested, 308 (33.2%) were rK39 RDT-positive. Spleen or bone marrow aspiration was done for 237 (77.2%) RDT-positive patients. Of these, 165 were confirmed parasitologically, yielding a positive predictive value of 69.6%. Only 126 (20.3%) of the 620 patients with a negative rK39 test underwent further testing by tissue aspiration, of which 22 (17.5%) were also parasitology positive. HIV test results were available for 570 (61.4%) patients and 36 (6.3%) were HIV-infected. Of the 187 parasitologically confirmed patients, 182 (97.3%) were started on VL treatment.

**Conclusions / Discussion:**

A negative rK39 test was often not followed by further testing and a positive rK39 test result was followed by tissue aspiration in three out of four cases. Further research is required to understand why the diagnostic work-up did not comply with the guidelines, including evaluating adherence to the VL clinical case definition and quality of rK39-RDT testing.

## Author Summary

The introduction of RDTs is one of the major advancements in leishmaniasis control programs. While the variability in performance from one endemic region to the other is well recognized, the utilization of these RDTs in the routine clinical setting has not been evaluated to date. In this study, we showed that the RDT use in routine practice setting has large deviations from the guidelines. Clinical suspicion of VL should be based on the full criteria of case definition as is recommended in the guidelines and the presence of the criteria should be checked by clinicians before requesting the RDT. Not respecting these clinical criteria can lead to low pretest probability of the disease and eventually low positive predictive value of the test used. Additionally, introducing regular monitoring activities with training and quality assurance system for leishmania RDTs is very important.

## Introduction

Visceral leishmaniasis (VL) is a vector borne, fatal disease caused by the protozoan parasite *Leishmania*. While *Leishmania donovani* is the etiologic agent in the East-African and Indian regions where transmission is anthroponotic, *L infantum* is the agent in the Mediterranean and Latin-American endemic regions, where transmission is zoonotic. It is an obligate intracellular organism that targets the reticuloendothelial system. The infection may initially remain dormant, manifesting in the presence of depressed cell mediated immunity at a later time. VL typically manifests with prolonged fever, weight loss, organomegaly and anemia [1].

Next to Sudan and South Sudan, Ethiopia has the highest VL burden in East-Africa. Moreover, North-West Ethiopia has the highest HIV co-infection rate in VL patients in the world, reaching up to 30–40% of those affected by VL [2,3]. Early diagnosis and treatment is one of the pillars of VL control. With regard to VL diagnostics, significant progress has been made over the last decade [4]. Rapid diagnostic tests (RDT) have been developed, most notably the rK39 RDT, which is cheap and easy to perform. While the diagnostic performance of this test was found to be consistently high in the Indian sub-continent, its sensitivity was suboptimal in the East-African region [5–19]. However, the RDTs have their well-known limitations, as they can also be positive in asymptomatically infected persons and in previously treated cases. Because antileishmanial antibodies wane only slowly after treatment, the rK39 RDT remains positive for several months to years after treatment, making the test useless to diagnose VL relapse in a patient who presents again with fever after an initial cure.

According to the current World Health Organization (WHO) and Ethiopian guidelines, in endemic areas clinically suspected patients (fever > 2 weeks, splenomegaly and weight loss) without history of VL should undergo testing with rK39 RDT. If positive, they should be treated for VL [20,21]. Indeed, the probability of VL in those who fulfill the clinical case definition and are rK39 positive was generally found to be high (a positive predictive value (PPV) > 90% at referral centers) [17]. However, in areas where the sensitivity of rK39 is less than 90% like in East-Africa [5], a negative test result should be followed by another test. This can be the direct agglutination test (DAT) or parasitological evaluation on tissue samples (WHO TRS 2010) [21]. The negative predictive value (NPV) of the rK39 test in east Africa was found to be 81% as compared to 95% in the Indian sub-continent [17]. The VL and the HIV guidelines also specify that all VL suspects should be tested for HIV since it affects the indicated diagnostic work-up and treatment [20,22].

The evidence-base of these diagnostic algorithms builds on several well-conducted high quality studies [23–25] that have led to national and international guidelines [20,21]. There is, however, surprisingly limited data on how these tests and algorithms are actually used in routine clinical practice settings. For instance, not adhering to the VL case definition could influence the overall performance of the algorithms. Second, quality assurance can be challenging in resource poor and remote VL endemic regions, with no guarantee of the quality of the RDTs circulating within national programs and their performance.

We performed a retrospective assessment on how the RDTs are used in clinical practice in a teaching hospital in Ethiopia. We used routinely collected data to assess the rK39 RDT utilization practices and alignment with recommendations in the guidelines. Amongst all individuals referred for rK39 testing in a VL research and treatment center in North-West Ethiopia, we describe the diagnostic work-up performed in relation to the actual rK39 RDT test result (and other key clinical factors), the proportion tested for HIV and the proportion initiated on VL treatment.

## Methods

### Ethics statement

Ethical clearance for the retrospective analysis of the routinely collected data was obtained from the UoG Institutional Review Board. The data was collected and handled maintaining the confidentiality of the subjects without including their names.

### Study setting

The Leishmaniasis Research and Treatment Center (LRTC) was founded in 2005 and is situated on the premises of the University of Gondar (UoG) Hospital in North-West Ethiopia. It is part of the Leishmaniasis East Africa Platform (LEAP), an international clinical research network that includes investigators from Sudan, Kenya and Uganda. LEAP conducts collaborative clinical trials aimed at improving the management of leishmaniasis patients in the region. Good Clinical Practice and Good Clinical Laboratory Practice compliant clinical trials have been conducted at the LRTC center since 2005 with the support of the Drugs for Neglected Diseases *initiative (*DND*i)*.

In addition to the clinical trials, the center also provides free diagnostic and treatment services for all types of *Leishmania* patients coming to the UoG Hospital. Patients may directly come to the center or they may be referred to the center either from the different units of the hospital (medical outpatient service, emergency unit and medical wards) or from other health institutions (health centers and district hospitals) in the catchment area.

The center has its own laboratory where most of the necessary investigations for *Leishmania* diagnosis and follow up are conducted. These include complete blood count, rK39 RDT, blood chemistry and microscopy of tissue aspirations. The rK39 RDT is done only in the laboratory of the center, and not in any of the other laboratories in the hospital. However, requests can be made from any of the departments and units in the hospital. There is no standardized rK39 RDT test request form; hand-written requests are made using different lab request formats. The main hospital of UoG has a general clinical laboratory, an HIV care/ART laboratory, an emergency services laboratory and a teaching and training laboratory.

### VL diagnostic work-up and patient flow

Patients suspected of VL are evaluated at the different units of the hospital (outpatient departments, emergency unit, medical and pediatric wards) and referred to the LRTC for the necessary procedures and laboratory tests related to leishmaniasis. Quite a number of patients present directly to the LRTC. Unless there is a history of previous VL (relapse), rK39-RDT testing (DiaMed-ITLEISH-Bio-Rad Laboratories) will be the initial diagnostic test, with additional testing according to the RDT result and clinical evaluation. The white cell count, hemoglobin and platelet counts are systematically done, HIV testing and counseling is offered to all as part of provider initiated testing and counseling (PITC). Some of the patients may be referred to the center after some or all of the laboratory work up is done while others undergo the clinical evaluation and all laboratory tests at the center. After the results of the rK39 tests are collected from the LRTC laboratory, physicians requesting the test from the different departments and units will make their decisions concerning the patients. This may be followed by further work-up for other diseases or linking to the LRTC for further confirmation of VL or VL treatment. The final treatment decision for VL is made by LRTC physicians.

The confirmatory test used for VL diagnosis is tissue aspiration either from the spleen or bone marrow. While the spleen is the primary choice for its better yield, bone marrow aspiration will be done in case of increased bleeding tendency, small or non-palpable spleen, presence of ascites or pregnancy. Giemsa stained smears are examined under high power oil immersion microscopy and parasite load grading performed according to the WHO guidelines [21].

All VL cases are admitted for treatment, mostly to the LRTC, while non-VL patients will be referred to the other units of the hospital for their respective medical condition. Occasionally, often on patient request, VL patients may be referred to other VL treatment centers. Anti-leishmanial drugs are dispensed from the research center pharmacy to patients admitted to the center as well as to the other wards of the hospital.

### Study design and population

We conducted a retrospective record analysis of routinely collected data including all patients who had an rK39-RDT done at the laboratory of the LRTC of UoG hospital for suspicion of primary VL between June 2012 and June 2013.

### Data sources, collection and analysis

Data were sourced from different registers of LRTC. This included the laboratory register, in which all individuals undergoing rK39 testing were entered and all laboratory tests were recorded; the ward patient registration book, recording all cases evaluated at the center; and the pharmacy dispensing log. Additional data was collected from the hospital laboratory register to complete the missing data for patients referred to the center after some investigations (hematology and HIV tests) at the hospital laboratory.

A standard format was prepared to collect the following information on all individuals that were tested with the rK39 test: rk39 test result, age, sex, full blood count result, tissue aspiration site and result, HIV status and treatment decision for VL. Data were entered into an excel spread sheet and transferred to STATA 12 for analysis. PPV and NPV of the rK39 RDT were calculated comparing with the tissue aspiration results.

## Results

Between June 2012 and June 2013, a total of 928 rK39 RDT tests were performed for clinical suspicion of VL. Most patients tested were male (89.2%), with a median age of 25 years (IQR 20–30). No data were available on the proportion of those 928 complying with the clinical case definition, because this was not routinely recorded. Out of 668 with a complete peripheral blood count result, 74 (11.1%) had a normal profile; 354 (53%) pancytopenia; 142 (21.3%) bicytopenia and 98 (14.7%) monocytopenia (Table 1). Leukopenia (white blood cell count <4500/μl) was found in 67.7% (457/675), anemia (hemoglobin <11g/dl) in 74.1% (501/676) and thrombocytopenia (platelet count < 150,000/μl) in 75.1% (503/670). In total, 308 (33.2%) had a positive rK39 RDT test result. Hematological abnormalities were more common in individuals testing rK39 positive (Table 1).

**Table 1:** Demographic and hematological profile of patients undergoing rK39 RDT testing for visceral leishmaniasis, Gondar, Ethiopia, 2012–2013.

Variable	rK39 positive	rK39 negative	Total
	n (%)	n (%)	n (%)
Total	308 (33.2)	620 (66.8)	928 (100)
Age in years (median, IQR) (n = 912)	24 (20–28)	25 (20–32)	25 (20–30)
Proportion of children (<15 years)	14 (4.6%)	40 (6.6%)	54 (5.9)
Male sex (n = 926)	298 (97.1)	528 (85.3)	826 (89.2)
WBC count			
> 4500	38 (12.3)	180 (29.0)	218 (23.5)
3000–4500	29 (9.4)	121 (19.5)	150 (16.2)
1500–3000	101 (32.8)	95 (15.3)	196 (21.1)
< 1500	81 (26.3)	30 (4.8)	111 (12.0)
Missing	59 (19.2)	194 (31.3)	253 (27.3)
Hemoglobin			
> 11 g/dL	37 (12.0)	138 (22.3)	175 (18.9)
7–11 g/dL	131 (42.5)	134 (21.6)	265 (28.6)
< 7 g/dL	83 (26.9)	153 (24.7)	236 (25.4)
Missing	57 (18.5)	195 (31.5)	252 (27.2)
Platelets			
> 150,000	41 (13.3)	126 (20.3)	167 (18.0)
50,000–150,000	124 (40.3)	201 (32.4)	325 (35.0)
< 50,000	82 (26.6)	97 (15.6)	179 (19.3)
Missing	61 (19.8)	196 (31.6)	257 (27.7)
Blood result			
Normal	12 (3.9)	62 (10.0)	74 (7.9)
Monocytopenia	19 (6.2)	79 (12.7)	98 (10.6)
Bicytopenia	42 (13.6)	100 (16.1)	142 (15.3)
Pancytopenia	172 (55.8)	183 (29.5)	355 (38.3)
Missing	63 (20.5)	196 (31.6)	259 (27.9)

IQR: interquartile range; RDT: rapid diagnostic test; WBC: white blood cells

**Fig 1:**
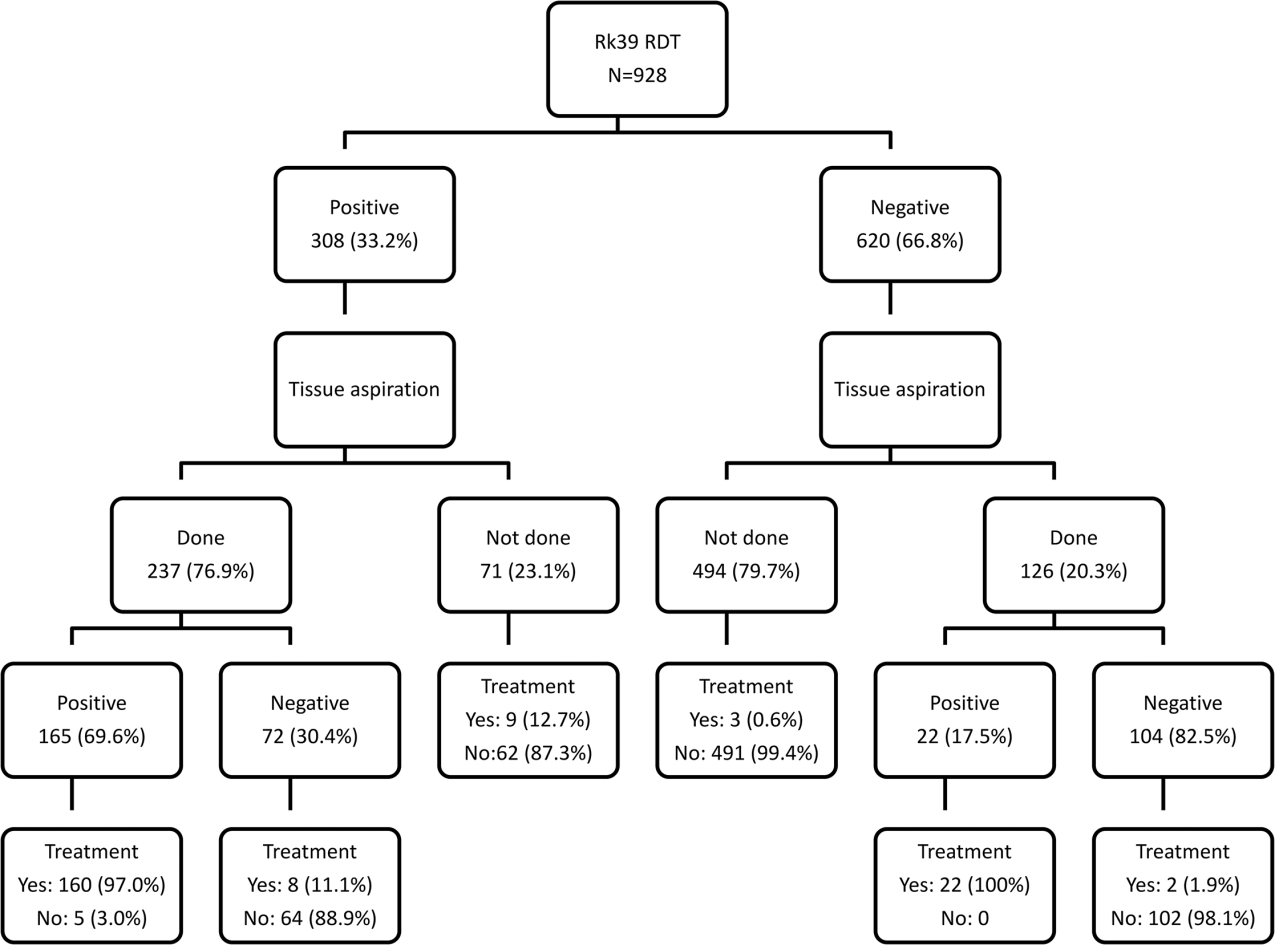
Flow diagram showing the diagnostic work-up of patients with suspected visceral leishmaniasis at the Leishmaniasis Research and Treatment Center, University of Gondar, Northwest Ethiopia.

Of the 308 rK39-positive individuals, 237 (77.2%) underwent tissue aspiration (139 from bone marrow and 225 from spleen), yielding a positive result in 165 (69.6%) (PPV 72.6% from bone marrow and 67.1% from spleen). There were 71 (32.8%) rK39-positive patients who did not undergo a tissue aspiration. Of the 620 individuals with a negative rK39 test result, 126 (20.3%) underwent tissue aspiration, which was positive in 22 (17.5%) (Fig 1 and Table 2). As many of the patients were referred for the test from different units outside of the LRTC, the subsequent diagnostic efforts made and (non-VL) treatments prescribed were not known for most of these patients from the available LRTC registers used.

**Table 2:** Tissue aspiration, HIV test results and treatment information of visceral leishmaniasis suspects, according to rK39 tests result, Gondar, Ethiopia, 2012–2013.

Variable	rK39 positive	rK39 negative
Total	308 (33.2%)	620 (66.8%)
Tissue aspiration done*	237 (76.9%)	126 (20.3%)
Bone marrow	95 (40.1%)	44 (34.9%)
Spleen	143 (60.3%)	82 (65.1%)
Positive result	165 (69.6%)	22 (17.5%)
Parasitology positive result	165 (69.6%)	22 (17.5%)
Spleen positive**	96 (67.1%)	14 (17.1%)
Bone marrow positive**	69 (72.6%)	8 (18.2%)
HIV testing recorded	183 (59.4%)	388 (62.6%)
HIV positive	15 (8.2%)	21 (5.4%)
Missing	125 (40.6%)	232 (37.4%)
VL treatment		
Treatment prescribed	177 (57.5%)	27 (4.4%)

* one patient had a bone marrow and a spleen aspirate

** percentages are calculated out of those who underwent the procedure

HIV test results were documented for 570 of the patients and 36 (6.3%) were HIV positive. The rK39-RDT was positive in 15 (41.7%) of the HIV positive patients, and 168 (31.5%) of the HIV negative group. Overall, 57.5% of the rK39-positive cases received VL treatment. Of the rK39 negative cases, 4.4% received VL treatment. There were a total of five parasitologically-confirmed cases that did not receive VL treatment at the LRTC. Three patients were referred to other treatment centers in the region; two patients disappeared after VL diagnosis.

## Discussion

RDTs have enabled an easy diagnosis of several diseases and have allowed to scale up treatment and control programs. However, there are concerns related to the high volume of tests used, the interpretation of results and quality assurance in the routine setting. This is one of the first studies evaluating VL testing practices by RDT in a routine clinical setting. While HIV test results and the subsequent actions for the rK39 negative patients were not available for a substantial proportion of individuals, linkage of the parasitologically confirmed VL cases to their treatment was good. The most striking finding was that the diagnostic work-up did not comply with the national guidelines [20]: a negative rK39 test was most of the time not followed by a second test for leishmanasis. On the other hand, a positive rK39 test result was followed by tissue aspiration in three out of four cases. The need for a parasitologically confirmed diagnosis in clinical trial protocols implemented at this clinical research center may partly explain the latter, but not the first scenario.

Recent data indicate a sensitivity of 87.2% (82.5–90.8%) and specificity of 96.4% (93.3–98.1%) of rK39 RDT testing (DiaMed-ITLEISH-Bio-Rad Laboratories) in East-Africa [19]. However, this was a case-control study where a negative DAT-result was used to define the negative controls; and the sensitivity and specificity may hence be overestimated. A recent Cochrane review estimated the pooled sensitivity of the rK39-RDT at 85% (95% CI 75 to 93%) and the pooled specificity at 91% (95% CI 80 to 97%) for the East African region (18). In most reported diagnostic studies from the region the prevalence of VL (pre-test probability) among clinical suspects was relatively high, ranging between 40 and 70% [17,25,26]. Consequently, the probability of VL in those testing positive in the rK39 test (the post-test probability of VL or the PPV) was typically above 90–95% [18]. In this regard, the probability of VL of 69.6% in those with a positive rK39 test found in this study was much lower than expected. Even if we would consider all the 71 rK39-positive patients that had not undergone tissue aspiration as true VL cases, the positive predictive value would still only be 76.6%.

Providing clear-cut answers as to why diagnostic practices in this hospital differed so much from recommendations on the guidelines will require additional research, and the same issues should address at other levels of the health system. However, we would like to present the following considerations—either related to the study population undergoing rK39 testing, or related to the rK39 test performance—that could potentially explain the findings, and which could be operating in parallel.

First, the VL diagnostic algorithm stipulates that testing should only be performed in individuals meeting the VL case definition [20]. More lenient testing practices (i.e. not strictly adhering to the VL case definition) would lead to a drop in VL pre-test probabilities and automatically also in post-test probabilities. It could also be that some patients with a repeat episode of fever after successful VL treatment might have been included in our series, which would reduce test specificity. Especially in a teaching hospital setting where rK39 testing can be requested from many different services and department, and by different types of health care providers, it is difficult to control strict adherence to the recommended indications for testing.

Due to the lack of a standardized laboratory request form for the rK39 test, (that should include essential clinical information), it is difficult to ascertain if the subjects tested with the rK39 really qualified or not in this retrospective analysis. All subjects to whom rK39 test is requested do not undergo reassessment at LRTC and subsequent linkage of the rK39 negative patients to the center is low. The test performance also depends on the characteristics of the population being tested and the spectrum and severity of underlying conditions [19,27]. The patient population in our study differed from those included in diagnostic accuracy study settings, especially in the sense that we hypothesize that the VL clinical case definition was not strictly applied.

A lower prevalence of VL in the population undergoing rK39 testing would automatically result in higher negative predictive values in those testing rK39 negative, possibly leading physicians to abstain from doing a follow-up test as per VL guidelines. However, this assumption cannot be verified and requires further study. A declining incidence of VL within Ethiopia may be an alternative explanation for a possibly lower pretest probability of VL, but there are so far no such indications at the national and regional level. Based on the monthly reports at the LRTC, the VL case load in the hospital has been stable over the last five years. The pattern of disease in the referral hospital setting may be another factor to consider. The region is also endemic for malaria, schistosomiasis, chronic viral hepatitis that mimic VL in their clinical presentation. Given the variety of complex and difficult to diagnose cases and atypical clinical manifestations seen at this health care level, physicians may request more tests to get additional laboratory diagnostic clues. The effect of HIV—affecting rK39 RDT test performance—could not be clearly assessed in our study, due to the small sample size and missing data on HIV status.

Besides less strict selection of the patients undergoing testing in routine clinical practice as compared to study settings, there might also be issues with the diagnostic performance of the tests used or with the test execution [27]. In many resource limited VL endemic regions, there is a lack of proper quality control and assurance systems covering all stages from evaluation of test performance, procurement to transportation and storage of the test kits used. While differences in performance between different brands have been well-documented, lot-to-lot variations could occur as well. Finally, national systems for training and supervision of rK39 test execution are non–existent.

We are aware that many alternative explanations or contributing factors might exist for the negative tissue aspirations in rK39-positive individuals, requiring further attention. For instance, the context of scarcity of drugs might encourage physicians to obtain a higher diagnostic certainty before starting VL treatment. Although only very few of the patients included in this study were actually enrolled in an ongoing clinical study, routine clinical practice in our center for patients directly presenting at the LRTC might still be influenced by the fact of being a clinical trial site. The tissue aspiration practices cannot be attributed to secondary financial gain by the clinic or the lab as these services are done for free. Finally, the suboptimal sensitivity of tissue aspiration might have contributed to the low PPV found in this study. Although substantial proportions (40%) of the aspirates were from bone marrow punctures, the diagnostic yield of spleen and bone marrow aspiration was comparable in this study. Last but not least, we should also verify a possible quality issue with parasitology readings at LRTC, as no test can ever be considered error-proof.

Importantly, the findings in our study do not necessarily reflect the reality at primary health care setting where midlevel health workers practice. At health center level tissue aspiration is not done and there is no other test available for further work-up. There are also limited supplies of diagnostics and medicine.

Pending further research, the following recommendations emerge from our study. First, adherence to the VL case definition during request for *Leishmania* tests should be assessed and enhanced [20,21]. Continuing medical education and supervision is indicated for health care professionals at all levels and hospital services to ensure a strict adherence to the VL case definition when using this RDT as a basis for a therapeutic decision. The design of a request form for rk39 RDT with tick boxes for the different components of the case definition could also be useful.

Second, quality assurance systems should be put in place that guarantee the quality of the diagnostic devices delivered at the health care facility, and the quality of the test execution and interpretation. Experiences of quality control and quality assurance systems designed for other diseases such as malaria can be adapted for leishmaniasis as well [19,27], and those should include all tests, RDT as well as microscopy.

Finally, national VL programs should establish data collection tools, allowing regular monitoring of testing practices and linkage to treatment, including treatment outcome monitoring. Such tools could function as an additional safety-check allowing the rapid identification of poorly performing RDTs. This is particularly important in settings where national VL control programs are still relatively weak and dependent on external actors for funding and/or delivery of tests. Moreover, there are now many different brands of rK39 RDT tests being marketed [19], as well as new RDTs based on different antigens (rK26, rK28). Not all of these have been appropriately evaluated in field settings or specific geographical regions.

In one of the major teaching hospitals in North-West Ethiopia, the diagnostic practices in VL do not fully comply with the national recommendations [20,21] in that a second leishmania test is not done for most of the rK39 negative suspects. The national treatment guidelines have a provision for a second test where rK39 tests are negative in highly suspected patients. Unfortunately a second test that can be deployed in the treatment centers is hard to find, as the DAT is costly and hard to deploy for routine testing. In addition, a positive rK39 test was followed by tissue aspiration in the majority of cases; and among those undergoing this procedure, three in ten were found to be parasite negative. This high rate of negativity by parasitology among rK39 positives can partly be explained by a false positive rK39 as well as by the inherent problems of microscopy. Further research is required to understand whether our findings in this reference center are generalizable to other levels of the health services. Besides evaluations of adherence to the VL case definition to guide rK39 testing, quality control measures for the rK39-RDT as well as parasitology should be established to ensure that VL diagnosis is carried out to the best interest of the patients. Availability of quality-assured rK39 for use in the national program and correct use and execution at the point of care are issues that deserve due attention.

## References

[1] van Griensven J, Diro E (2012) Visceral leishmaniasis. Infect Dis Clin North Am 26: 309–322. S0891-5520(12)00014-1 [pii]; 10.1016/j.idc.2012.03.005 22632641

[2] Hurissa Z, Gebre-Silassie S, Hailu W, Tefera T, Lalloo DG et al (2010) Clinical characteristics and treatment outcome of patients with visceral leishmaniasis and HIV co-infection in northwest Ethiopia. Trop Med Int Health 15: 848–855. TMI2550 [pii]; 10.1111/j.1365-3156.2010.02550.x 20487426

[3] Mengistu G, Ayele B (2007) Visceral Leishmaniasis and HIV co-infection in patients admitted to Gondar university hospital, northwest Ethiopia. Ethiopian Journal of Health Development 53–60.

[4] Srividya G, Kulshrestha A, Singh R, Salotra P (2012) Diagnosis of visceral leishmaniasis: developments over the last decade. Parasitol Res 110: 1065–1078. 10.1007/s00436-011-2680-1 22065060

[5] Chappuis F, Rijal S, Soto A, Menten J, Boelaert M (2006) A meta-analysis of the diagnostic performance of the direct agglutination test and rK39 dipstick for visceral leishmaniasis. BMJ 333: 723 bmj.38917.503056.7C [pii]; 10.1136/bmj.38917.503056.7C 16882683 PMC1592383

[6] Diro E, Techane Y, Tefera T, Assefa Y, Kebede T et al. (2007) Field evaluation of FD-DAT, rK39 dipstick and KATEX (urine latex agglutination) for diagnosis of visceral leishmaniasis in northwest Ethiopia. Trans R Soc Trop Med Hyg 908–914. 10.1016/j.trstmh.2007.05.002 17624385

[7] Canavate C, Herrero M, Nieto J, Cruz I, Chicharro C et al (2011) Evaluation of two rK39 dipstick tests, direct agglutination test, and indirect fluorescent antibody test for diagnosis of visceral leishmaniasis in a new epidemic site in highland Ethiopia. Am J Trop Med Hyg 84: 102–106. 84/1/102 [pii]; 10.4269/ajtmh.2011.10–0229 21212210 PMC3005501

[8] Singh DP, Goyal RK, Singh RK, Sundar S, Mohapatra TM (2010) In search of an ideal test for diagnosis and prognosis of kala-azar. J Health Popul Nutr 28: 281–285. 20635639 10.3329/jhpn.v28i3.5557 PMC2980893

[9] Rouf MA, Rahman ME, Islam MN, Islam MN, Ferdous NN et al (2009) Sensitivity, specificity and predictive values of immunochromatographic strip test in diagnosis of childhood kala-azar. Mymensingh Med J 18: S1–S5. 19377416

[10] Boelaert M, El-Safi S, Hailu A, Mukhtar M, Rijal S et al (2008) Diagnostic tests for kala-azar: a multi-center study of the freeze-dried DAT, rK39 strip test and KAtex in East Africa and the Indian subcontinent. Trans R Soc Trop Med Hyg 102: 32–40. S0035-9203(07)00287-8 [pii]; 10.1016/j.trstmh.2007.09.003 17942129

[11] Sundar S, Singh RK, Maurya R, Kumar B, Chhabra A et al (2006) Serological diagnosis of Indian visceral leishmaniasis: direct agglutination test versus rK39 strip test. Trans R Soc Trop Med Hyg 100: 533–537. S0035-9203(05)00330-5 [pii]; 10.1016/j.trstmh.2005.08.018 16325874

[12] Chappuis F, Mueller Y, Nguimfack A, Rwakimari JB, Couffignal S et al (2005) Diagnostic accuracy of two rK39 antigen-based dipsticks and the formol gel test for rapid diagnosis of visceral leishmaniasis in northeastern Uganda. J Clin Microbiol 43: 5973–5977. 43/12/5973 [pii]; 10.1128/JCM.43.12.5973–5977.2005 16333084 PMC1317204

[13] Boelaert M, Rijal S, Regmi S, Singh R, Karki B et al (2004) A comparative study of the effectiveness of diagnostic tests for visceral leishmaniasis. Am J Trop Med Hyg 70: 72–77. 14971701

[14] Chappuis F, Rijal S, Singh R, Acharya P, Karki BM et al (2003) Prospective evaluation and comparison of the direct agglutination test and an rK39-antigen-based dipstick test for the diagnosis of suspected kala-azar in Nepal. Trop Med Int Health 8: 277–285. 1026 [pii]. 12631320 10.1046/j.1365-3156.2003.01026.x

[15] Veeken H, Ritmeijer K, Seaman J, Davidson R (2003) Comparison of an rK39 dipstick rapid test with direct agglutination test and splenic aspiration for the diagnosis of kala-azar in Sudan. Trop Med Int Health 8: 164–167. 996 [pii]. 12581443 10.1046/j.1365-3156.2003.00996.x

[16] Zijlstra EE, Nur Y, Desjeux P, Khalil EA, El-Hassan AM et al (2001) Diagnosing visceral leishmaniasis with the recombinant K39 strip test: experience from the Sudan. Trop Med Int Health 6: 108–113. tmi680 [pii]. 11251906 10.1046/j.1365-3156.2001.00680.x

[17] Boelaert M, Verdonck K, Menten J, Sunyoto T, van GJ et al (2014) Rapid tests for the diagnosis of visceral leishmaniasis in patients with suspected disease. Cochrane Database Syst Rev 6: CD009135. 10.1002/14651858.CD009135.pub2 []. PMC4468926 24947503

[18] ter Horst R, Tefera T, Assefa G, Ebrahim AZ, Davidson RN et al (2009) Field evaluation of rK39 test and direct agglutination test for diagnosis of visceral leishmaniasis in a population with high prevalence of human immunodeficiency virus in Ethiopia. Am J Trop Med Hyg 80: 929–934. 80/6/929 [pii]. 19478251

[19] Cunningham J, Hasker E, Das P, El SS, Goto H et al (2012) A global comparative evaluation of commercial immunochromatographic rapid diagnostic tests for visceral leishmaniasis. Clin Infect Dis 55: 1312–1319. cis716 [pii]; 10.1093/cid/cis716 22942208 PMC3478143

[20] Federal Ministry of Health, Ethiopia (2013) Guidelines for diagnosis, treatment and prevention of leishmaniasis in Ethiopia, 2nd edition.

[21] World Health Organization (3-22-2010) Control of the Leishmaniases. WHO Technical Report Series 949: Report of a meeting of the WHO Expert Committee on the Control of Leishmaniases, Geneva, 22–26 March 2010 Geneva, Swetzerland: World Health Organization.

[22] Federal HIV/AIDS prevention and control office and Federal Minsitry of Health (2007) Guidelines for management of opportunistic infections and antiretroviral treatment in adolescents and adults in Ethiopia.

[23] Mueller YK, Kolaczinski JH, Koech T, Lokwang P, Riongoita M et al (2014) Clinical epidemiology, diagnosis and treatment of visceral leishmaniasis in the Pokot endemic area of Uganda and Kenya. Am J Trop Med Hyg 90: 33–39. ajtmh.13-0150 [pii]; 10.4269/ajtmh.13-0150 24218406 PMC3886423

[24] Sundar S, Chakravarty J (2012) Recent advances in the diagnosis and treatment of kala-azar. Natl Med J India 25: 85–89. 22686715

[25] Chappuis F, Rijal S, Singh R, Acharya P, Karki BM et al (2003) Prospective evaluation and comparison of the direct agglutination test and an rK39-antigen-based dipstick test for the diagnosis of suspected kala-azar in Nepal. Trop Med Int Health 8: 277–285. 1026 [pii]. 12631320 10.1046/j.1365-3156.2003.01026.x

[26] Ritmeijer K, Melaku Y, Mueller M, Kipngetich S, O'keeffe C et al (2006) Evaluation of a new recombinant K39 rapid diagnostic test for Sudanese visceral leishmaniasis. Am J Trop Med Hyg 74: 76–80. 74/1/76 [pii]. 16407349

[27] World Health Organization and UNICEF/UNDP/World Bank/WHO Special Programme for Research and Training in Tropical Diseases (2011) Visceral leishmaniasis rapid diagnostic performance, Diagnostic evaluation series No. 4.

